# *hTERT* promoter polymorphism, -1327C>T, is associated with the risk of epithelial cancer

**DOI:** 10.1186/2193-1801-2-249

**Published:** 2013-05-31

**Authors:** Toshihiko Iizuka, Motoji Sawabe, Kaiyo Takubo, Miao Liu, Yukio Homma, Motofumi Suzuki, Tomio Arai

**Affiliations:** Research Team for Geriatric Pathology, Tokyo Metropolitan Institute of Gerontology, 35-2 Sakae-cho, Itabashi-ku, Tokyo, 173-0015 Japan; Department of Diagnostic Pathology, National Center for Global Health and Medicine, 1-21-1, Toyama, Shinjuku-ku, Tokyo, 162-8655 Japan; Bioresource Center for Geriatric Research, Tokyo Metropolitan Geriatric Hospital, 35-2 Sakae-cho, Itabashi-ku, Tokyo, 173-0015 Japan; Section of Molecular Pathology, Graduate School of Health Care Science, Tokyo Medical and Dental University, Bunkyo, Tokyo, Japan; Department of Urology, The University of Tokyo, 7-3-1 Hongo, Bunkyo-ku, Tokyo, 113-8655 Japan; Department of Cardiovascular and Renal Research Laboratory, Virginia Commonwealth University, Richmond, VA USA; Department of Pathology, Tokyo Metropolitan Geriatric Hospital, 35-2 Sakae-cho, Itabashi-ku, Tokyo, 173-0015 Japan

**Keywords:** Telomere, Telomerase, *hTERT*, Cancer susceptibility, Genetic polymorphism

## Abstract

Telomeres are repetitive nucleotide sequences that cap the end of eukaryotic chromosomes. Attrition of these structures has been associated with carcinogenesis in many tissues, and therefore, they are essential for chromosome stabilization. Telomeres are maintained by telomerase complexes, of which human telomerase reverse transcriptase (hTERT) is an essential component. A functional polymorphism, -1327C>T (rs2735940), located in the promoter of the *hTERT* gene is associated with telomere length in peripheral blood leukocytes. We hypothesized that this polymorphism might affect susceptibility to various epithelial malignancies. The -1327C>T polymorphism was examined in 1,551 consecutive autopsy cases (mean age, 80.3 years), and we focused on its effect on the risks of overall and each primary malignancies. The polymorphism was further studied in 391 clinical prostate cancer patients who were diagnosed via prostate biopsy, using autopsy cases as controls. In the autopsy cases, the risk of epithelial malignancy, after adjusting for age, sex, smoking, and drinking habits, was significantly lower for the TT genotype than the CC (reference) genotype (adjusted odds ratio = 0.61, 95% CI = 0.42-0.90). Among primary malignancies, latent prostate cancer, colorectal cancer, and lung cancer were the most strongly associated with the polymorphism. In the study using clinical prostate cancer patients, susceptibility to clinical prostate cancer was lower for -1327 T carriers than for -1327 T non-carriers, but this finding was not significant. The data suggest that the *hTERT* promoter polymorphism, -1327C>T, is an independent factor influencing the risk of various epithelial malignancies in elderly Japanese.

**Electronic supplementary material:**

The online version of this article (doi:10.1186/2193-1801-2-249) contains supplementary material, which is available to authorized users.

## Background

Telomeres are special structures at the end of chromosomes in eukaryotic cells that are essential for the chromosome stabilization. Erosion of telomeres leads to chromosomal instability, i.e., formation of end-to-end fusions, degradation, and rearrangement of chromosomes (Greider 1991). Telomerase is a ribonucleoprotein complex that elongates telomeres by adding TTAGGG nucleotide repeats, thus maintaining telomere integrity. Human telomerase-reverse transcriptase (hTERT) is the rate limiting subunit of the telomerase complex and the expression level of *hTERT* is primarily regulated at the transcription level (Ducrest et al. 2002).

Telomere attrition can enhance the early stages of carcinogenesis (Hackett and Greider 2002), and the shortening of telomeres can increase genetic instability and tumor formation in mice (Blasco et al. 1997; Rudolph et al. 1999; Chin et al. 1999). Short telomeres are also prevalent in precursors of many epithelial cancers in humans (Meeker et al. 2004; Kammori et al. 2007; Aida et al. 2010). Moreover, dysfunctional telomeres and mutations in genes that encode the telomerase complex have been described in congenital dyskeratosis, a human disease that confers an increased risk of developing certain cancers (Vulliamy and Dokal 2008). Patients with congenital dyskeratosis are 11 times more susceptible to cancer than the general population (Alter et al. 2009).

Human telomere length is characteristic of each individual: a subject with long telomeres in one organ generally has long telomeres in other organs (Takubo et al. 2002). Differences in telomere length among individuals are attributable, to a large extent, to genetic factors (Slagboom et al. 1994), raising the possibility that genetic polymorphisms influence cancer susceptibility through telomere length.

Matsubara et al. reported that an *hTERT* -1327C>T (rs2735940) polymorphism within the promoter region, a T/C transition 1327 bp upstream of the transcription start site, affects transcriptional activity (Matsubara et al. 2006). In their study, telomeres in the peripheral blood leukocytes of -1327 T allele carriers were significantly longer than those of -1327 T non-carriers. Because changes in telomere length can affect cancer susceptibility, we hypothesized that the -1327C>T polymorphism might affect susceptibility for various malignancies. To test this hypothesis, we examined the relationship between the -1327C>T polymorphism and malignancy susceptibility in autopsy cases and in patients with clinical prostate cancer.

## Results

### Characterization of the autopsy subjects

Of the 1,551 subjects, 960 had at least one malignancy. The malignancies included gastric cancer (177 cases), lung cancer (167 cases), colorectal cancer (147 cases), leukemia (104 cases), prostate cancer (103 cases), and malignant lymphoma (81 cases). In 89 of 103 prostate cancer cases, the disease was first diagnosed at autopsy (i.e., latent cancer) and found before death (i.e., clinical cancer) in the remaining 14 cases. All of the malignancies (epithelial and non-epithelial) found in the autopsy cases are listed in Additional file 1: Table S1. The 591 cases with no malignancies were used as a control group in subsequent statistical analyses. Clinical features of the control group and malignant cases are shown in Table 1 and Additional file 2: Table S2 (analysis stratified by sex). Although the mean age of all subjects was 80.3 years, malignant cases were significantly younger than the control group (p = 0.0001), especially in female (p < 0.0001). The male/female ratio of all subjects was 1.19 and was significantly higher in cases with malignancy compared to the control group (p = 0.0031). Smoking rate was significantly higher in cases with malignancy than in the control group (p = 0.028). Alcohol drinking rate was not significantly different between cases with malignancy and control group.

**Table 1:** **Characterization of the autopsy cases**

Clinical features	Control^a^ (n = 591)	Cases with malignancy (n = 960)	Total (n = 1551)	p-value
Age (years, average ± SD)	81.4 ± 9.2	79.6 ± 8.6	80.3 ± 8.9	0.0001^b^
Male/Female ratio	0.98	1.34	1.19	0.0031^c^
Smoker / Total^d^	0.45	0.51	0.49	0.028^c^
Alcohol drinker / Total^e^	0.23	0.27	0.25	0.074^c^

^a^Cases with no malignancy.

^b^p-value based on one-way ANOVA.

^c^p-value based on chi-square analysis.

^d^Calculated for cases whose smoking history was available (n = 1391). Smokers were defined as those who smoked one or more cigarettes per day.

^e^Calculated for cases whose drinking history was available (n = 1433). Alcohol drinkers were defined as those who consumed 15 grams or more alcohol per day.

### Characterization of the -1327C>T *hTERT* genotype in autopsy subjects

\The genotypic frequencies of -1327C>T were 44% for CC, 45% for CT, and 11% for TT. The minor allelic frequency was 34%, and the allele distribution was consistent with Hardy-Weinberg equilibrium (p = 0.79). The genotypic distribution was similar to a previous finding for the Japanese population (Matsubara et al. 2006). There were no significant differences in the distribution of age, sex, and smoking and drinking habits among the genotype groups (Additional file 3: Table S3).

### Association between the -1327C>T *hTERT* genotype and the risk of overall malignancy in autopsy cases

Table 2 shows the genotype distribution in cases with no malignancy, single malignancy and multiple malignancies. Crude analysis did not uncover a significant association between genotype and the risk of having at least one malignancy. However, adjustment for age, sex, smoking status and alcohol consumption revealed a significantly lower risk for the TT genotype compared with CC (adjusted OR = 0.68, 95% CI = 0.48–0.98). In the additive model, the T-allele was associated with a lower risk of malignancy (adjusted OR = 0.70, 95% CI = 0.51–0.98). The risk of multiple malignancies was significantly lower for the CT and TT genotypes compared with CC (crude OR = 0.69, 0.48; 95% CI = 0.51–0.95, 0.27–0.82; respectively), an association that was unchanged following adjustment (adjusted OR = 0.68, 0.46; 95% CI = 0.48–0.95, 0.25–0.79). In the dominant, recessive and additive models, the T-allele was associated with a lower risk of multiple malignancies (crude OR = 0.65, 0.58, 0.69; 95% CI = 0.48–0.87, 0.33–0.96, 0.55–0.87; respectively); after adjustment the association remained unchanged (adjusted OR = 0.63, 0.55, 0.67; 95% CI = 0.45–0.86, 0.31–0.93, 0.53–0.86).

**Table 2:** **Association between the -1327C>T*hTERT*genotype and the risk of overall malignancy in autopsy cases**

	Genotype distribution, n (%)			Risk for malignancy^a^				Risk for multiple malignancies^b^			
	Control^c^	Single malignancy cases	Multiple malignancy cases	Crude OR (95% CI)	p-value	Adjusted OR^d^ (95% CI)	p-value	Crude OR (95% CI)	p-value	Adjusted OR^d^ (95% CI)	p-value
CC	245 (41.5)	308 (43.3)	130 (52.2)	1 (reference)		1 (reference)		1 (reference)		1 (reference)	
CT	272 (46.0)	324 (45.6)	100 (40.2)	0.87	0.22	0.86	0.22	**0.69**	**0.021**	**0.68**	**0.024**
				(0.70 - 1.08)		(0.68 - 1.09)		**(0.51 - 0.95)**		**(0.548- 0.95)**	
TT	74 (12.5)	79 (11.1)	19 (7.6)	0.74	0.085	**0.68**	**0.041**	**0.48**	**0.0065**	**0.46**	**0.0046**
				(0.53 - 1.04)		**(0.48 - 0.98)**		**(0.27 - 0.82)**		**(0.25 - 0.79)**	
*Dominant model*											
CC	245 (41.5)	308 (43.3)	130 (52.2)	1 (reference)		1 (reference)		1 (reference)		1 (reference)	
CT + TT	346 (58.5)	403 (56.7)	119 (47.8)	0.84	0.11	0.82	0.087	**0.65**	**0.0043**	**0.63**	**0.0040**
				(0.69 - 1.04)		(0.66 - 1.03)		**(0.48 - 0.87)**		**(0.45 - 0.86)**	
*Recessive model*											
CC + CT	517 (87.5)	632 (88.9)	230 (92.4)	1 (reference)		1 (reference)		1 (reference)		1 (reference)	
TT	74 (12.5)	79 (11.1)	19 (7.6)	0.79	0.16	0.73	0.080	**0.58**	**0.033**	**0.55**	**0.024**
				(0.58 - 1.10)		(0.53 - 1.04)		**(0.33 - 0.96)**		**(0.31 - 0.93)**	
*Additive model*^e^				0.86	0.063	**0.70**	**0.036**	**0.69**	**0.0017**	**0.67**	**0.0014**
				(0.74 - 1.01)		**(0.51 - 0.98)**		**(0.55 - 0.87)**		**(0.53 - 0.86)**	

The risk of malignancy was estimated by calculating crude OR and OR adjusted for age, sex, smoking status and alcohol habit using a logistic regression model in autopsy cases (n = 1551). Significant associations highlighted in bold.

*OR* odds ratio, *CI* confidence interval.

^a^Cases with at least one malignancy were compared with control.

^b^Cases with more than two malignancies were compared with control.

^c^Cases with no malignancy (n = 591).

^d^Calculated for cases for whom smoking and drinking history was available (n = 1371).

^e^Applied by including the number of T-alleles (0,1,2) as a continuous variable in the logistic regression model.

### Association between the -1327C>T *hTERT* genotype and the risk of epithelial/non-epithelial malignancies in autopsy cases

An association between -1327C>T *hTERT* and malignancy risk was further analyzed by dividing the tumors into epithelial and non-epithelial classes (Table 3). In the crude analysis, the risk of epithelial malignancy was significantly lower for TT compared with CC (crude OR = 0.65; 95% CI = 0.45–0.93), an association that was unchanged after adjustment (adjusted OR = 0.61; 95% CI = 0.42–0.90). In the dominant and additive models, the T-allele was associated with a lower risk of epithelial malignancy (crude OR = 0.79, 0.81; 95% CI = 0.64–0.98, 0.69–0.96; respectively), which remained unchanged after adjustment (adjusted OR = 0.78, 0.80; 95% CI = 0.62–0.98, 0.67–0.95). In contrast, -1327C>T *hTERT* was not significantly associated with the risk of developing a non-epithelial malignancy. Since two types of cancer (epithelial and non-epithelial) were tested, Bonferroni correction was performed. The association was still significant after the correction, where a significance level of 0.05/2 = 0.025 was used (TT vs CC, and additive model).

**Table 3:** **Association between the -1327C>T*hTERT*genotype and the risks of epithelial/non-epithelial malignancy in autopsy cases**

Genotype	Genotype distribution, n(%)			Risk of epithelial malignancy^a^				Risk of non-epithelial malignancy^b^			
	Control^c^	Epithelial malignancy	Non-epithelial malignancy	Crude OR (95% CI)	p-value	Adjusted OR^d^ (95% CI)	p-value	Crude OR (95% CI)	p-value	Adjusted OR^d^ (95% CI)	p-value
CC	245 (41.5)	372 (47.2)	100 (42.6)	1 (reference)		1 (reference)		1 (reference)		1 (reference)	
CT	272 (46.0)	343 (43.5)	102 (43.4)	0.83	0.11	0.83	0.13	0.92	0.61	0.90	0.58
				(0.66 - 1.04)		(0.65 - 1.06)		(0.66 - 1.27)		(0.63 - 1.29)	
TT	74 (12.5)	73 (9.3)	33 (14.0)	**0.65**	**0.020**	**0.61**	**0.012**	1.09	0.71	0.94	0.80
				**(0.45 - 0.93)**		**(0.42 - 0.90)**		(0.68 - 1.74)		(0.55 - 1.56)	
*Dominant model*											
CC	245 (41.5)	372 (47.2)	100 (42.6)	1 (reference)		1 (reference)		1 (reference)		1 (reference)	
CT + TT	346 (58.5)	416 (52.8)	135 (57.4)	**0.79**	**0.033**	**0.78**	**0.033**	0.96	0.77	0.91	0.58
				**(0.64 - 0.98)**		**(0.62 - 0.98)**		(0.70 - 1.30)		(0.65 - 1.27)	
*Recessive model*											
CC + CT	517 (87.5)	715 (90.7)	202 (86.0)	1 (reference)		1 (reference)		1 (reference)		1 (reference)	
TT	74 (12.5)	73 (9.3)	33 (14.0)	0.71	0.054	**0.68**	**0.033**	1.14	0.56	0.98	0.95
				(0.51 - 1.01)		**(0.47 - 0.97)**		(0.73 - 1.76)		(0.60 - 1.59)	
*Additive model*^e^											
				**0.81**	**0.012**	**0.80**	**0.0096**	1.01	0.94	0.95	0.67
				**(0.69 - 0.96)**		**(0.67 - 0.95)**		(0.81 - 1.26)		(0.74 - 1.21)	

The risk of malignancy was estimated by calculating crude OR and OR adjusted for age, sex, smoking status and alcohol habit using a logistic regression model in autopsy cases (n = 1551).

Significant associations highlighted in bold. *OR* odds ratio, *CI* confidence interval.

^a^Cases with epithelial malignancy were compared with control.

^b^Cases with non-epithelial malignancy were compared with control.

^c^Cases with no malignancy (n = 591).

^d^Calculated for cases for whom smoking and drinking history was available (n = 1371).

^e^Applied by including the number of T-alleles (0,1,2) as a continuous variable in the logistic regression model.

### Association between the -1327C>T *hTERT* genotype and the risks of primary malignancies of various origins in autopsy cases

The association between -1327C>T and the risks of malignancies of various origins was analyzed. Genotypic frequencies are shown for each type of cancer in Additional file 4: Table S4. The association between *hTERT* genotype and susceptibility to malignancy was particularly strong for colorectal, lung and latent prostate cancers (Table 4).

**Table 4:** **Association between the −1327 C>T*hTERT*genotype and the risks of various types of malignancies in autopsy cases**

Genotype	Lung cancer^a^ (n = 167)				Colorectal cancer^a^ (n = 147)				Latent prostate cancer^a^ (n = 89)			
	Crude OR (95% CI)	p-value	Adjusted OR^b^ (95% CI)	p-value	Crude OR (95% CI)	p-value	Adjusted OR^b^ (95% CI)	p-value	Crude OR (95% CI)	p-value	Adjusted OR^b^ (95% CI)	p-value
CC	1 (reference)		1 (reference)		1 (reference)		1 (reference)		1 (reference)		1 (reference)	
CT	0.92	0.67	0.97	0.88	0.91	0.64	0.87	0.50	**0.57**	**0.030**	**0.56**	**0.046**
	(0.65 - 1.32)		(0.65 – 1.44)		(0.63 - 1.33)		(0.58 – 1.30)		**(0.34 - 0.95)**		**(0.31 – 0.99)**	
TT	**0.47**	**0.022**	**0.48**	**0.038**	**0.38**	**0.0081**	**0.39**	**0.0012**	**0.38**	**0.025**	**0.27**	**0.0083**
	**(0.23 - 0.90)**		**(0.23 – 0.96)**		**(0.16 - 0.79)**		**(0.17 – 0.82)**		**(0.14 - 0.89)**		**(0.078 – 0.73)**	
*Dominant model*^c^	0.83	0.28	0.86	0.44	0.80	0.23	0.76	0.17	**0.53**	**0.0090**	**0.49**	**0.0088**
	(0.59 - 1.17)		(0.59 – 1.26)		(0.56 - 1.15)		( 0.51 – 1.12)		**(0.33 - 0.85)**		**(0.28 – 0.84)**	
*Recessive model*^d^	**0.49**	**0.024**	**0.49**	**0.032**	**0.40**	**0.0085**	**0.42**	**0.015**	0.49	0.089	**0.35**	**0.030**
	**(0.24 - 0.91)**		**(0.24 – 0.94)**		**(0.17 - 0.81)**		**(0.18 – 0.86)**		(0.18 - 1.11)		**(0.10 – 0.91)**	
*Additive model*^e^	0.78	0.066	0.80	0.12	**0.74**	**0.037**	**0.72**	**0.032**	**0.60**	**0.0060**	**0.54**	**0.0033**
	(0.60 - 1.02)		(0.59 – 1.06)		**(0.56 - 0.98)**		**(0.53 - 0.97)**		**(0.40 - 0.87)**		**(0.35 – 0.82)**	

The risk of each type of malignancy was estimated by calculating crude OR and OR adjusted for age, sex, smoking status and alcohol habit using a logistic regression model in autopsy cases (n = 1551). Significant associations highlighted in bold.

*OR* odds ratio, *CI* confidence interval.

^a^ Cases with each type of malignancy were compared with control (cases with no malignancy).

^b^Calculated for cases for whom smoking and drinking history was available (n = 1371).

^c^CT + TT vs CC.

^d^TT vs CT + CC.

^e^Applied by including the number of T-alleles (0,1,2) as a continuous variable in the logistic regression model.

The risk of lung cancer was significantly lower for cases with TT genotype compared with CC, before (crude OR = 0.47, 95% CI = 0.23–0.90) and after (adjusted OR = 0.48, 95% CI = 0.23–0.96) adjustment. In the recessive model, the T-allele was associated with a lower risk of lung cancer before (crude OR = 0.49, 95% CI = 0.24–0.91) and after (adjusted OR = 0.49, 95% CI = 0.24–0.94) adjustment.

The risk of colorectal cancer was significantly lower for cases with TT genotype compared with CC, before (crude OR = 0.38, 95% CI = 0.16–0.79) and after (adjusted OR = 0.39, 95% CI = 0.17–0.82) adjustment. In the recessive and additive models, the T-allele was associated with a lower risk of colorectal cancer before (crude OR = 0.40, 0.74; 95% CI = 0.17–0.81, 0.56–0.98)) and after (adjusted OR = 0.42, 0.72; 95% CI = 0.18–0.86, 0.53–0.97) adjustment.

The crude risk of latent prostate cancer was significantly lower for cases with the CT and TT genotype compared to CC (crude OR = 0.57, 0.38; 95% CI = 0.34–0.95, 0.14–0.89); adjustment did not alter this association (adjusted OR = 0.56, 0.27; 95% CI = 0.31–0.99, 0.078–0.73). In the dominant and additive models, theT-allele was associated with a lower risk of latent cancer before (crude OR = 0.53, 0.60; 95% CI = 0.33–0.85, 0.40–0.87; respectively) and after (adjusted OR = 0.49, 0.54; 95% CI = 0.28–0.84, 0.35–0.82) adjustment.

When Bonferroni-corrected significance level (0.05 divided by the number of cancer types tested, 0.05/11 = 0.0045) were used, the association for all cancer types were insignificant. However, this correction might be too conservative, because the risks for each type of malignancy are supposed to be not independent.

The occurrence of other malignancies, including leukemia, malignant lymphoma, and gastric, breast, hepatocellular, pancreatic and bile duct carcinomas, did not reveal significant associations with the *hTERT* genotype before Bonferroni correction (data not shown).

### -1327C>T *hTERT* genotype and the risk of clinical prostate cancer

Because only 14 cases of clinical prostate cancer were included in the 1,551 autopsy cases, the polymorphism was further studied in 391 patients diagnosed by prostate biopsy before death. Residence-matched autopsy cases of 323 males who did not have cancer were used as control subjects. After adjusting for age by logistic regression analysis, the risk of developing clinical prostate cancer was lower in cases with a T-allele than in cases without it, but the differences were not significant (Table 5). No association was revealed after stratifying the subjects by clinical stage or Gleason score (data not shown).

**Table 5:** **Association between the -1327C>T*hTERT*genotype and the risk of clinical prostate cancer**

Genotype	Genotype frequency distribution, n (%)		Crude OR	p-value	OR adjusted for age	p-value
	Clinical prostate cancer patients	Control	(95% CI)		(95% CI)	
CC	186 (48.1)	137 (43.1)	1 (reference)		1 (reference)	
CT	157 (40.6)	142 (44.6)	0.81	0.20	0.77	0.14
			(0.59 - 1.12)		(0.54 - 1.09)	
TT	44 (11.4)	39 (12.3)	0.83	0.45	0.83	0.52
			(0.51 - 1.35)		(0.48 - 1.44)	
*Dominant model*						
CC	186 (48.1)	137 (43.1)	1 (reference)		1 (reference)	
CT + TT	201 (51.9)	181 (56.9)	0.83	0.21	0.79	0.16
			(0.61 - 1.13)		(0.56 - 1.10)	
*Recessive model*						
CC + CT	343 (88.6)	279 (87.7)	1 (reference)		1 (reference)	
TT	44 (11.4)	39 (12.3)	0.92	0.71	0.95	0.85
			(0.58 - 1.46)		(0.57 - 1.60)	
*Additive model*^a^			0.88	0.25	0.86	0.25
			(0.71 - 1.10)		(0.68 - 1.11)	

Clinical prostate cancer patients (n = 391) were compared with residence-matched male autopsy cases without malignancy (n = 323).

Genotype data were not available for 4 and 5 patients respectively.

*OR* odds ratio, *CI* confidence interval.

^a^Applied by including the number of T-alleles (0,1,2) as a continuous variable in the logistic regression model.

## Discussion

Matsubara et al. reported that the T allele in the *hTERT* promoter polymorphism, -1327C>T, is associated with longer telomeres in the peripheral blood leukocytes. The presence of the T allele in the promoter corresponds to higher transcriptional activity of the gene (Matsubara et al. 2006). In this study, an examination of 1,551 autopsy cases found that the T allele is also associated with a lower risk of epithelial malignancy, particularly prostate (latent), colorectal and lung cancers. The polymorphism was also evaluated in 391 clinical prostate cancer patients using autopsy cases as controls. Although the risk of clinical prostate cancer was also lower for -1327 T carriers than for non-carriers, the difference was not significant.

Telomere-shortening has been associated with epithelial malignancy in humans and in animal models, but the relationship has not been observed for non-epithelial cancers. In humans, the spectrum of malignancies varies considerably between pediatric and adult ages (DePinho 2000). The majority of malignancies in children are non-epithelial, while epithelial malignancies predominate in adults, as chromosomal instability induced by telomere attrition is enhanced with age. Non-epithelial malignancies are primarily found in p53 mutant mice. However, if the mice also have dysfunctional telomeres, epithelial malignancies predominate with non-reciprocal translocations (Artandi et al. 2000). In the current study, the -1327 T-allele was associated with a significantly lower risk of epithelial malignancies, which was not observed with non-epithelial malignancies. These data are consistent with the premise that telomere-shortening is associated with epithelial, but not non-epithelial, carcinogenesis.

Telomere-shortening is thought to occur early in the initiation of epithelial malignancies (Meeker et al. 2004) because telomere attrition is prevalent in many types of precancerous lesions (Meeker 2006; Aida et al. 2010; Kammori et al. 2007). It was reported that the telomere length in the precursor lesion of prostate adenocarcinoma (high-grade prostatic intraepithelial neoplasia) is shorter than in normal epithelium (Meeker et al. 2002). Telomere length was also reportedly shortened in adenomas of the colon (O’Sullivan et al. 2006). Since the T-allele has higher transcriptional activity, these previous observations and our results suggest that the -1327 T allele of *hTERT* reduces the risk of cancer through its higher telomere-elongation capacity.

The association between the -1327C>T polymorphism and cancer susceptibility has been controversial. Savage et al. reported that -1327 T is associated with reduced risk of breast cancer in individuals with a family history of breast cancer (Savage et al. 2007), but this observation was not confirmed by Varadi et al. (Varadi et al. 2009). Furthermore, Choi et al. reported that the -1327 T-allele is associated with an increased risk of lung cancer (Choi et al. 2009), which is contrary to the findings of this study. Although it is difficult to explain the discrepancies, they might be partly due to differences in the age of the subjects. In our analysis, the mean age of the subjects was 80.3 years, whereas it was 61.3 years in the Choi et al. study. Telomeres can have both suppressive and facilitative effects on cancer development, depending on the stage of carcinogenesis (Hackett and Greider 2002). Telomere shortening can increase genetic instability and tumor formation in the early stage of carcinogenesis. Conversely, in later stages, telomerase activation and telomere function can also facilitate tumor progression by stabilizing the genomes of cancer cells and conferring the capacity for immortal growth. Since telomeres shorten with age, it seems reasonable that the predominant effect of telomere attrition (cancer-facilitative or suppressive) depends on the age of the subjects. Another possible explanation is the difference in the genetic background. Multiple genetic loci contribute in concert to the risk of cancer and the opposing effects (flip-flop phenomenon) of the -1327 T on cancer risk may be explained by interactive effects or linkage disequilibrium (Lin et al. 2007).

Recently, there are several reports which show that polymorphisms in the *hTERT* gene and in *TERT-CLPTM1L* locus are associated with the risks of various cancer types. Interestingly, these polymorphisms are also associated with telomere length (Nan et al. 2011; Melin et al. 2012; Bojesen et al. 2013; Lan et al. 2013). These findings are consistent with the hypothesis that *hTERT* polymorphisms can affect cancer risk through the effect on telomere length.

-1327 T was associated with significantly lower risk of latent prostate cancer, and the risk of clinical prostate cancer was also lower for -1327 T carriers, but it was not significant. The prevalence rate of latent prostate cancer is much higher than that of clinical prostate cancer, especially in Asians (Ruijter et al. 1999). The vast majority of latent prostate cancer does not progress to clinical prostate cancer, and the risk factors for latent and clinical cancer are thought to be different. Our results suggest that the -1327 T>C polymorphism is more important for carcinogenesis of latent prostate cancer than that of clinical prostate cancer, and it is consistent with the hypothesis that telomere attrition is involved in the early stage of carcinogenesis.

There are a number of limitations to our study. One is that data on telomere length are lacking. A report by Martinez and Blasco suggested that *hTERT* is involved in carcinogenesis through non-telomerase activity (Martinez and Blasco 2011). To confirm that -1327C>T is affecting cancer risk through its telomere-elongation capacity, telomere length in the epithelial cells of various tissues should be examined. The second limitation is the sample size of the study. Overall epithelial malignancy was significantly associated with -1327C>T, even after Bonferroni correction. However, the association between the genotype and the risk for each type of malignancy was significant only for latent prostate cancer, colorectal cancer and lung cancer. Furthermore, the risks for such cancer types were insignificant after Bonferroni correction. To confirm the risk in each type of malignancy, further studies involving larger numbers of each type of epithelial malignancy are needed. The third limitation is about the character of the study subjects. We cannot rule out the possibility of selection bias, since the autopsy subjects are from single geriatric hospital and the average age was very old (80.3 years). However, the cause of death of our subjects was similar to that of general Japanese population, and in addition, the -1327C>T genotype frequencies were similar to those reported in other Japanese population study, which suggest that selection bias would be minor.

In spite of these limitations, our data provide valuable information on the role of *hTERT* in carcinogenesis of the elderly. Most previous studies on the association between *hTERT* polymorphism and cancer risk have been performed using relatively younger population. Since telomere shortens with age, the effect of *hTERT* polymorphism on cancer risk may depend on age. Further studies using diverse age group subjects are necessary.

## Conclusion

This study presented evidence that the -1327C>T polymorphism in the *hTERT* promoter may be associated with susceptibility for epithelial malignancies, especially prostate cancer (latent), colorectal and lung cancer in the elderly Japanese population. The results warrant further investigation with a larger number of cases of each type of malignancy, and with a wide variation in ages.

## Methods

### Autopsy subjects

The group of autopsy subjects consisted of 1,551 consecutive cases (843 males and 708 females) from Tokyo Metropolitan Geriatric Hospital, a community-based geriatric hospital in Tokyo, that were registered in the Internet-based Database of Japanese SNPs for Geriatric Research (JG-SNP) (Sawabe et al. 2004). Autopsies were performed between 1995 and 2004 on 40% of patients who died at the hospital, regardless of the cause of death. The major cause of death was malignant disease, coronary heart disease and pneumonia in 33%, 20% and 13% of the subjects, respectively. This proportion were similar to the causes of death reported in a survey conducted by the Ministry of Health, Labor and Welfare of Japan (Vital statistics of Japan 2000), where the cause of death in about 30% of the population was malignant disease. These results indicate that the selection bias would be minor for the recruited subjects. The ages of the subjects ranged from 46 to 104 years, with a mean age of 80.3 years. Subjects were classified as smokers (including ex-smokers) versus non-smokers, and alcohol drinkers versus non-drinkers based on histories obtained from medical records. Information on tobacco-smoking and alcohol use was unavailable for 160 and 118 subjects, respectively. Smokers were defined as those who smoked one or more cigarettes per day. Alcohol-drinkers were defined as those who consumed 15 grams or more alcohol per day. The percentage of smokers was 49%, while that of alcohol drinkers was 25%. Written informed consent was obtained from the family of each subject at the time of autopsy. The study protocol for autopsy cases was approved by the Ethical Committee of the Tokyo Metropolitan Geriatric Hospital.

### Subjects for the analysis of clinical prostate cancer

Study participants were Japanese patients with clinical prostate cancer treated by the Department of Urology at the University of Tokyo Hospital or at its affiliated hospital located in the Kanto area of Japan. Characteristics of the 391 patients have been reported elsewhere (Liu et al. 2009). Adenocarcinoma of the prostate was pathologically confirmed by prostate biopsy in all cases. Patients with a family history of prostate cancer were excluded from this study. The mean age ± SD of the clinical cancer patients was 70.8 ± 8.0 years (range, 48 to 89 years). The clinical stages of the tumors were T1 or T2 for 167 patients and T3 or T4 for 24 patients. The Gleason scores of the tumors were ≥8 for 150 patients and ≤ 7 for 241 patients. The study protocol was conducted with the approval of the Ethics Committee of the University of Tokyo and the internal review board of each of the affiliated hospitals. Written informed consent was obtained from each patient prior to their enrollment in the study. The controls consisted of 323 residence-matched male autopsy cases at the Tokyo Metropolitan Geriatric Hospital. Pathological examinations confirmed that none of the control cases had suffered from malignancies. The mean age ± SD of patients in the control group was 79.2 ± 9.2 years (range, 49 to 100 years). There was a significant difference in the ages of the 391 clinical subjects and the 323 controls; therefore, logistic regression analysis was used to adjust for age.

### Assessment of the number of primary malignancies

Each subject was evaluated for primary malignancies using autopsy findings and medical records. Only histologically diagnosed malignancies were counted. When one subject had multiple malignant lesions, they were considered to be of multiple or single origin depending on pathological findings and clinical information.

### Genotyping

Genomic DNA was extracted from the renal cortex (of autopsy cases) or from the peripheral blood specimen (of patients with clinical prostate cancer) by the phenol-chloroform method. Genotyping of the -1327C>T (rs2735940) SNP in the *hTERT* gene was conducted with a TaqMan assay using an ABI PRISM 7000 Sequence Detection System (Applied Biosystems, Foster City, CA, USA) in accordance with the manufacturer’s instructions. The sequences of the *hTERT*-specific primers were 5′-ACAATTCACAAACACAGCCCTTTAAAA-3′ (forward) and 5′-CCCTCCCTGGGCTGTCA-3′ (reverse). The sequence of the TaqMan reporter probe was 5′-CTTAGGATTAC[G/A]GGTCGCT-3′. The PCR reaction was performed in a 20-μl volume using TaqMan Universal PCR Master Mix, No AmpErase UNG (ABI). Each well contained 10 ng of genomic DNA. The cycling protocol was as follows: initial denaturation at 95°C for 10 min, followed by 40 cycles of 92°C for 15 sec and 60°C for 1 min. Genotype could be determined in 99.3% of cases.

### Statistical analysis

Statistical analysis was performed using JMP version 9.0.0 (SAS Institute Inc., Cary, NC, USA). Chi-square tests were conducted to examine the Hardy-Weinberg equilibrium, to compare sex ratios, smoking status (smoker or non-smoker) and drinking status (drinker or non-drinker) between the genotypes. The odds ratio (OR) and 95% confidence interval (CI) adjusted for age, sex, smoking status, and drinking status were calculated using an unconditional logistic regression model when estimating the risk of malignancy for each genotype. The risks of overall malignancy and each primary malignancy were evaluated. Co-dominant, dominant, recessive and additive models were applied in the analysis. All tests were 2-tailed, with statistical significance set at p < 0.05. Bonferroni correction was performed by dividing the significance level (0.05) by the number of cancer types tested. In the analysis using clinical prostate cancer patients, the OR was adjusted using only age as covariant, because histories on smoking and alcohol drinking were not available for the patients. However, confounding effects of smoking /drinking would be minor because there is no evidence, to our knowledge, which definitely shows that smoking /drinking is associated with the risk of prostate cancer.

## Electronic supplementary material

Additional file 1: Table S1: Malignancies in autopsy cases. (XLS 9 KB)

Additional file 2: Table S2: Characterization of the autopsy cases (analysis stratified by sex). (XLS 10 KB)

Additional file 3: Table S3: Characterization of the autopsy cases of each -1327C>T *hTERT* genotype. (XLS 10 KB)

Additional file 4: Table S4: Genotype frequencies in autopsy cases with each type of malignancy. (XLS 10 KB)
